# Periodic Oscillations in Daily Reported Infections and Deaths for Coronavirus Disease 2019

**DOI:** 10.1001/jamanetworkopen.2020.17521

**Published:** 2020-08-17

**Authors:** Qasim Bukhari, Yusuf Jameel, Joseph M. Massaro, Ralph B. D’Agostino, Sheraz Khan

**Affiliations:** 1 McGovern Institute for Brain Research, Massachusetts Institute of Technology, Cambridge; 2 Department of Civil and Environmental Engineering, Massachusetts Institute of Technology, Cambridge; 3 Department of Mathematics and Statistics, Boston University, Boston, Massachusetts; 4 Department of Radiology, Massachusetts General Hospital, Harvard Medical School, Boston; 5 Athinoula A. Martinos Center for Biomedical Imaging, Massachusetts General Hospital, Harvard Medical School, Massachusetts Institute of Technology, Boston

## Abstract

This cross-sectional study investigates oscillatory patterns in daily reported infections and deaths for coronavirus disease 2019.

## Introduction

Severe acute respiratory syndrome coronavirus 2 has affected millions of people worldwide. The trend of coronavirus disease 2019 (COVID-19) cases is not similar across countries, with several countries experiencing a decrease in the daily reported cases and deaths, while several others are reporting a surge in the daily reported cases and deaths. Studies of prior epidemics^1,2^ have suggested oscillatory patterns and cyclicity when analyzing long-term (ie, decades) epidemiological data. However, to our knowledge, high-frequency oscillations (ie, weekly) have not been reported during prior epidemics. In this cross-sectional study, we investigate oscillatory patterns in COVID-19 cases and deaths.

## Methods

This study was not submitted for institutional review board approval and informed consent was not sought because it uses publicly available data at the population level, in accordance with 45 CFR §46. The underlying methods in this analysis are described in detail in the eAppendix in the Supplement. In brief, we obtained the daily new cases and deaths for COVID-19 between February 29 and July 2, 2020, from Worldometer,^2^ applied a 3-day moving average to remove high-frequency fluctuations in the daily new cases and deaths, and then performed spectral analysis and calculated the phase-lag between daily reported cases and deaths. Data analysis was performed in July 2020 with Python statistical software version 3.7 (Python) and open-source signal-processing toolboxes, as described in the eAppendix in the Supplement.

## Results

We identified oscillatory patterns in the daily reported new cases and deaths with a periodicity of approximately 1 week for the US, Germany, Canada, Italy, Brazil, and the United Kingdom (Figure). The data from Germany and Italy show dampened oscillations (decreasing amplitude with time) for both newly reported infections and deaths, with a −92% change in peak-to-peak oscillatory pattern in the daily reported deaths in Germany between April and July 2020, which might indicate a substantial decay in the spread of the virus. However, the data from the US and Brazil show no sign of dampening, with a −43% change in peak-to-peak oscillatory pattern in the daily reported deaths in the US between April and July 2020, which suggests that the US and Brazil are still not at the decaying phase. The spectral density plot in panel B of the Figure confirms an oscillatory pattern of 7 days. The rose plot in panel C of the Figure shows the polar histogram of the phase angle difference between daily new cases and deaths and demonstrates a lag between daily new cases and deaths of 2 days for the US and 1 day for Germany. However, this lag is not due to the epidemiology of the disease but possibly is associated with bias in the surveillance system.

**Figure.**
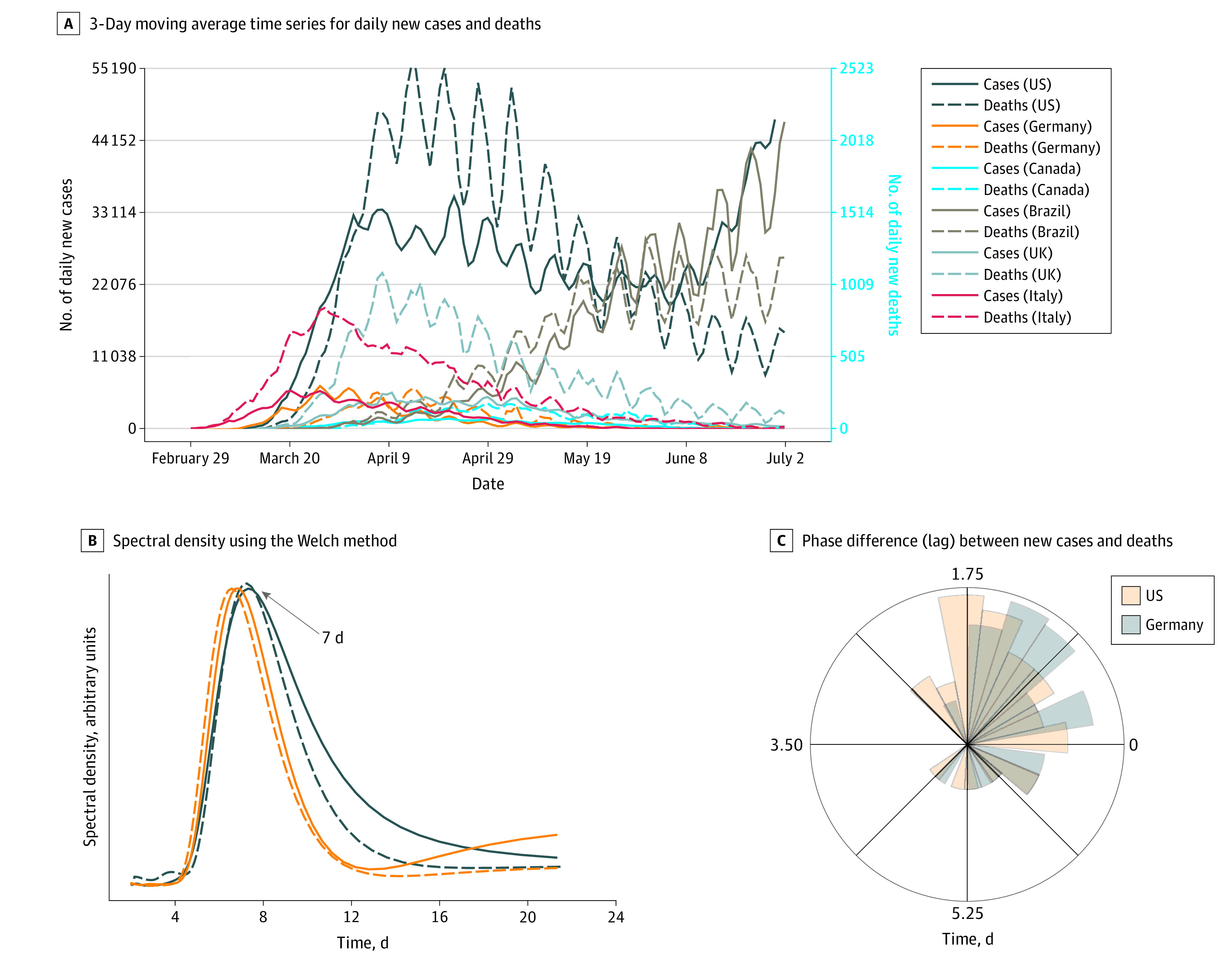
Oscillation Patterns for Coronavirus Disease 2019 Cases and Deaths in the US, Germany, Canada, Brazil, UK, and Italy A, Three-day moving average time series for daily new cases and deaths. B, Spectral density calculated using the Welch method. C, Phase difference (lag) between new cases and deaths in the US and Germany.

## Discussion

It is possible that these periodic oscillations in daily reported cases are associated with testing bias, with higher rates of testing during certain days of a week. However, these periodic oscillations were also observed for positive test rates,^2^ suggesting that other variables, such as epidemiological or social factors leading to higher transmission on certain days, might be associated with these oscillations. Interestingly, periodic oscillations in new cases have been observed in multiple models for the spread of infectious diseases. For instance, consistent seasonal oscillations have been reported for smallpox in Japan, India, and Sweden in data sets that span several decades.^3,4^ Importantly, these oscillations arise naturally from the model instead of a periodic forcing term or other exogenous factors. Spatiotemporal oscillations have also been reported in large data sets for dengue hemorrhagic fever from Thailand, suggesting that immune interactions between the serotypes might play a role in the observed pattern.^5^ These oscillations should be included in the estimation of the effective reproduction number (R_t_), similar to the way seasonality is accounted for in influenza. We urge the scientific community to conduct an in-depth exploration of the periodicity in COVID-19 cases and deaths, which might lead to improved COVID-19 predictions and understanding of the transmission of the disease.

## Notes

Supplement. **eAppendix.** Supplementary Methods **eReferences.**
