# Supplementary material for: Periodic Oscillations in Daily Reported Infections and Deaths for Coronavirus Disease 2019
Source: JAMA Netw Open. 2020 Aug 17;3(8):e2017521. doi: 10.1001/jamanetworkopen.2020.17521 (PMC7431996; doi:10.1001/jamanetworkopen.2020.17521)
Supplement: Supplement. — eAppendix. Supplementary Methods eReferences. [file jamanetwopen-3-e2017521-s001.pdf]

Supplementary Online Content

Bukhari Q, Jameel Y, Massaro JM, D’Agonstino RB Sr, Khan S. Periodic oscillations in daily reported infections and deaths for coronavirus disease 2019. *JAMA Netw Open*. 2020;3(8):e2017521. doi:10.1001/jamanetworkopen.2020.17521

eAppendix. Supplementary Methods

eReferences.

This supplementary material has been provided by the authors to give readers additional information about their work.

© 2020 Bukhari Q et al. *JAMA Network Open.*

eAppendix. Supplementary Methods

Data Retrieval

The data for the Figure were programmatically retrieved from Worldometer (https://www.worldometers.info/coronavirus/country/) using python package Beautiful Soup (https://pypi.org/project/beautifulsoup4/).

Moving Average Filter

To remove high frequency fluctuations in the daily new cases and deaths, we applied the moving average filter [1] of three days. Moving average is the most common filter in signal processing and operates by averaging a number of points from the input signal $x(t)$ to produce each point in the output signal $y(t)$. Mathematically:

$$y(t) = \frac{1}{M}\sum_{i=0}^{M} x(t+i)$$

Where M is the number of points in the moving average.

Spectral Analysis

The Power Spectral Density in panel B of the Figure was computed using the Welch method [2] with a Hanning window of 20 days as implemented in SciPy.[3]

Filtering

The resulting time series were band-pass filtered in the periodicity of oscillation identify by the welch method using mne-python. [4]

Hilbert Transform

The analytic signal $\hat{Y}(t)$ was calculated by combining the filtered time series with its Hilbert transform [5] into a complex time series as implemented in SciPy [3]:

$$z(t) = z_r(t) \; + \; jz_i(t) \; = y(t) + j\,\mathcal{H}[y(t)]$$

Where $\mathcal{H}$ represents the Hilbert Transform operation

The resulting time series $z(t)$ can be seen as a rotating vector in the complex plane whose length corresponds to the envelope of the original time series $y(t)$ and whose phase grows according to the dominant frequency. The instantaneous phase angle was computed in the complex plane as:

$$\phi(t) = \; \angle\, z(t) = arctan\left[\frac{z_i(t)}{z_r(t)}\right]$$

© 2020 Bukhari Q et al. *JAMA Network Open.*

Rose Plot

The rose plot in panel C of the Figure represents the polar histogram of phase angle difference between daily new cases and deaths, mathematically:

$$\Delta\,\phi(t) = \phi_{new\_cases}(t) - \phi_{new\_deaths}(t)$$

Code and Data Availability

The code and data API for reproducing our analysis reported in this article are available at https://github.com/SherazKhan/covid_oscillations.

eReferences

1. Smith, Steven W. "The scientist and engineer's guide to digital signal processing." (1997): 35.
2. P. Welch, "The use of the fast Fourier transform for the estimation of power spectra: A method based on time averaging over short, modified periodograms", IEEE Trans. Audio Electroacoust. vol. 15, pp. 70-73, 1967.
3. Virtanen, Pauli, et al. "SciPy 1.0: fundamental algorithms for scientific computing in Python." Nature methods 17.3 (2020): 261-272.
4. Gramfort, Alexandre, et al. "MEG and EEG data analysis with MNE-Python." Frontiers in neuroscience 7 (2013): 267.
5. Oppenheim, Alan V. Discrete-time signal processing. Pearson Education India, 1999.

© 2020 Bukhari Q et al. *JAMA Network Open.*
